# Selective Head Cooling and NOX Inhibition Protect the Blood–Brain Barrier in Neonatal Epilepsy

**DOI:** 10.3390/antiox14121454

**Published:** 2025-12-03

**Authors:** Helena Parfenova, Jianxiong Liu, Shyamali Basuroy, Rong Zhang, Mimily Harsono, Massroor Pourcyrous

**Affiliations:** Departments of Physiology and Pediatrics, University of Tennessee Health Science Center, Memphis, TN 38163, USA; jliu22@uthsc.edu (J.L.); sbasuroy535@gmail.com (S.B.); zhangrong0812@163.com (R.Z.); mharsono@uthsc.edu (M.H.); mpourcyrous@uthsc.edu (M.P.)

**Keywords:** endothelial dysfunction, oxidative stress, antioxidants, hypothermia, setanaxib, sulforaphane, epilepsy

## Abstract

Epileptic seizures in the neonatal brain induce oxidative stress and disrupt the blood–brain barrier (BBB), leading to long-term cerebrovascular and neurodevelopmental deficits. This study examined the protective effects of selective head cooling and NADPH oxidase (NOX) inhibition on BBB integrity following seizures. Neonatal seizures were induced in newborn pigs with bicuculline under normothermic or selective head cooling conditions. BBB disruption was assessed by Evans Blue extravasation and quantification of circulating brain-derived endothelial cells (CD45^−^/CD146^+^/GluT1^+^). Seizures under normothermia caused marked BBB leakage, cerebrovascular apoptosis, and elevated endothelial biomarkers, whereas selective head cooling (cortical temperature ~25 °C, body ~35 °C) significantly reduced these effects. Pharmacological inhibition of NOX with setanaxib (5 mg/kg) or sulforaphane (0.4 mg/kg) also prevented BBB disruption during normothermia. In vitro, primary porcine and human brain endothelial cells exposed to glutamate or TNF-α showed increased NOX activity, ROS production, apoptosis, and barrier leakage, all attenuated by NOX inhibitors or moderate hypothermia (<30 °C). These findings identify endothelial NOX as a key mediator of seizure-induced BBB injury and demonstrate that both NOX inhibition and selective head cooling effectively preserve cerebrovascular integrity. Combined hypothermic and antioxidant therapy may offer a promising strategy to prevent cerebrovascular injury and BBB damage in neonatal epilepsy.

## 1. Introduction

Epileptic seizures are harmful for the developing brain and produce life-long neurocognitive deficits [1,2,3,4,5,6]. Numerous causes of neonatal seizures include hypoxic–ischemic insults, intraventricular hemorrhage, infections, and metabolic and genetic abnormalities [2,5,6]. Our studies in newborn pigs have demonstrated that epileptic seizures cause cerebral vascular injury and long-term loss of endothelium-mediated regulation of cerebral blood flow [7,8,9,10]. Accumulated evidence in epileptic patients also suggests that seizures cause cerebral vascular injury [11,12,13,14,15]. Oxidative stress triggered by excitotoxic neurotransmitters and inflammatory mediators is a major driver of cerebral vascular injury and neuronal death caused by seizures. The key contribution of oxidative stress to cerebral vascular injury is supported by studies in newborn pigs [8,10,16] and other models [17]. Blood–brain barrier (BBB) failure following epileptic seizures has been demonstrated in both animal and human studies [18,19,20,21]. In turn, BBB damage may contribute to self-reinforcing seizures thereby worsening neuroinflammation and secondary brain injury, ultimately driving epilepsy progression [22,23,24,25,26]. This highlights the importance of the BBB as a potential therapeutic target for epilepsy.

Uncovering the mechanisms that protect BBB integrity is critical in the prevention of neonatal encephalopathy caused by seizures. Despite growing interest in using a wide spectrum of antioxidants, no significant progress in BBB protection has been achieved. Our previous work uncovered the role of selective head cooling as an efficient approach to prevention of cerebral vascular disorders in neonates with epileptic seizures [27]. However, the role of head cooling on BBB integrity in epilepsy has not yet been investigated.

The NADPH oxidase (NOX) family, composed of NOX1-NOX5 isoforms, is a potent source of reactive oxygen species (ROS) production in the brain, acting as an upstream factor that controls oxidative stress and neuroinflammation [28,29,30,31]. High constitutive expression of Nox4 appears to be a specific characteristic of vascular cells in systemic and cerebral circulation [28,31,32,33]. Our previous work using siRNA-mediated gene silencing on the NOX family members in cerebral vascular endothelial cells identified NOX4 as the predominant isoform responsible for increased ROS production in response to glutamate and TNF-α, which are excitotoxic and inflammatory stressors relevant to seizures [32,33].

The present study aims to investigate the effects of hypothermia induced by selective head cooling on BBB permeability under oxidative stress conditions associated with epileptic seizures. We will test three key hypotheses: (1) hypothermia attenuates BBB disruption in both an in vivo neonatal epilepsy model and an in vitro BBB model exposed to seizure-relevant pro-oxidants; (2) hypothermia preserves BBB integrity by inhibiting endothelial NOX activity; and (3) under normothermic conditions, pharmacological inhibitors of NOX prevent BBB damage induced by oxidative stress. Elucidating the mechanisms that protect BBB integrity is essential for developing strategies to mitigate seizure-induced neonatal encephalopathy.

## 2. Materials and Methods

### 2.1. Animals

Newborn pigs (1–5 days old, 1.5–3.0 kg, either sex) were purchased from a commercial breeder. The University of Tennessee Health Science Center (UTHSC) Animal Care and Use Committee (IACUC) reviewed and approved all procedures involving animals in compliance with National Institutes of Health (NIH) Guide for the Care and Use of Laboratory Animals. Overall, 90 pigs were used for this study. All experiments were conducted according to the ARRIVE guidelines 2.0 for animal research.

### 2.2. Model of Neonatal Epileptic Seizures

Bicuculline, the GABA_A_ receptor antagonist, induces glutamatergic epileptic seizures by disrupting the balance between inhibitory and excitatory neurotransmitters. Newborn pigs were initially anesthetized with ketamine-xylazine (33:2 mg/kg, i.m.) and maintained by administration of α-chloralose (50 mg/kg i.v.) as described previously [8,10]. Pigs were intubated, ventilated with room air, and instrumented to monitor vital signs and blood gases. Pigs were paralyzed by pancuronium bromide (0.2 mg/kg, i.v.). The body temperature was maintained at 37–38 °C by a servo-controlled heating pad. Three groups of epileptic pigs included the normothermia Group I and two selective head cooling groups, including Group II (preventive head cooling) and Group III (therapeutic head cooling). Two additional groups of normothermic newborn pigs were used to investigate the effects of NOX inhibitors on the outcome of epileptic seizures. The selective NOX1/NOX4 inhibitors setanaxib GKT137831 (5 mg/kg i.p.) or non-selective NOX inhibitor sulforaphane (SFN, 0.4 mg/kg i.p.) were administered to normothermic pigs 30 min prior to bicuculline administration. Bicuculline (3 mg/kg, i.p.) produced a burst of epileptiform discharges and cerebral hyperemia that lasted in all groups for ~2 h [27]. Selective head cooling did not reduce the bicuculline-induced epileptiform activity [27]. After recovery from seizures and anesthesia, pigs were extubated and transferred to the animal care facility for 24–48 h.

### 2.3. Selective Head Cooling in Epileptic Newborn Pigs

In this study, we used the selective head cooling model previously established in our laboratory [27]. Selective head cooling was achieved by placing two cotton towel-wrapped ice packs on both sides of the intact scalp over the parietotemporal region as described earlier [27]. Rectal temperature was measured using the YSI tele-thermometer model 43TA (Yellow Spring Instrument Co, Yellow Springs, OH, USA). Applying ice packs directly over the skull allows selective reduction in cortical brain temperature to approximately 24–25 °C (moderate hypothermic), while the core body temperature remains within the mild hypothermic range (34–35 °C). Mild hypothermia (34–35 °C) is considered safe for neonates and is widely used in clinical practice as a standard of care for neonatal hypoxic–ischemic encephalopathy brain injury and associated seizures. A steady state reduction in core body temperature (34–35 °C) indicating mild hypothermia was achieved within 30 min following the head ice pack placement. Brain cortex temperature reduced to 25.6 ± 0.3 °C within 30 min of selective head cooling and maintained at this level during 2.5 h of seizures [27]. Seizures were induced by bicuculline (3 mg/kg, i.p.) in two groups of hypothermic pigs. For preventive head cooling (Group II), the head ice packs were placed 30 min before seizure induction. For therapeutic head cooling (Group III), the head packs were placed 20 min after seizures induction. In both experimental groups II and III, the hypothermia intervention was sustained during the ictal phase, which lasted approximately 2 h. Our previous study in newborn pigs demonstrated that selective head cooling during the ictal period prevented cerebrovascular injury in newborn pigs but did not reduce seizure activity [27]. A detailed analysis of bicuculline-evoked electroencephalographic (EEG) parameters in normothermic newborn pigs and in pigs subjected to selective head cooling demonstrated that head cooling did not suppress EEG amplitude, spectral power, spike frequency distribution, or seizure duration [27].

During the postictal state, the head ice packs were removed, and pigs were rewarmed by heating pads. Core body temperature returned to normothermia level (37–38 °C) in 2–3 h. Pigs were extubated and transferred to the animal care facility for recovery for 24–48 h.

### 2.4. In Vivo BBB Permeability in Epileptic Newborn Pigs

Extravasation of albumin-bound Evans Blue (EB) is a common indicator for analyzing in vivo BBB breakdown in various models of neurological disease [34,35]. EB is a non-cell permeable azo dye which binds to serum albumin producing a fluorescent complex. Following intravenous injection, albumin-bound EB remains in the bloodstream unless a defect in the structural integrity of the BBB has occurred. Appearance of the fluorescent EB signal in the vascular-free brain parenchyma allows quantitative analysis of BBB leakage. To evaluate BBB permeability, sham control (Group I) and postictal pigs (Groups II and III) were anesthetized 4, 24, or 48 h after recovery, intubated, instrumented with a vein catheter, and ventilated with room air. Evans Blue (4 mL/kg) reconstituted in saline (2%) was infused via a vein catheter at the rate of 1 mL per min. Following a 1-h period for Evans Blue circulation, animals were injected with heparin (1000 U). Transcardial perfusion was performed with lactated Ringer’s solution containing 0.4% heparin for 30 min to remove residual intravascular dye and stored in cold phosphate-buffered saline at +4 °C overnight. The cerebral cortex was dissected into four major parts: frontal, parietal, temporal, and occipital lobes. Two pieces from each lobe (2 g wet tissue) were homogenized in PBS (1:1.5 *v*/*v*). Evans Blue was extracted from the brain cortex by homogenization with 100% trichloroacetic acid (1:1 *v*/*v*) [34]. The tissue extract was cleared by centrifugation, dispensed in triplicates onto 96-well Costar black assay plate, and mixed with 100% EtOH (1:3 *v*/*v*). Protein-bound Evans Blue fluorescence (excitation/emission peaks, 620/680 nm) was measured by the Synergy HT multi-mode microplate reader (BioTek Instruments, Winooski, VT, USA). The standard curve was constructed by serial dilutions of the Evans Blue solution containing TCA and EtOH (linear ranges of detection: 20–1000 ng/mL). The fluorescence intensity was normalized to the tissue weight in each sample.

### 2.5. Brain-Derived Circulating Endothelial Cells (BCECs) in Peripheral Blood of Newborn Pigs

Peripheral blood mononuclear cells (PMNCs) from venous blood of newborn pigs were separated by Histopaque 1077 density gradient centrifugation as described [9]. The PMNC-enriched interface layer was collected by syringe, diluted 1:10 with Dulbecco’s Phosphate-Buffered Saline (DPBS), and pelleted for 10 min at 1200× *g*. BCECs were immunodetected as CD45-negative PMNCs co-expressing CD31 (common endothelial cell marker) and Glut1 (BBB endothelial marker) (CD45^−^/CD31^+^/Glut1^+^) [9].

### 2.6. Primary Porcine and Human Cerebral Microvascular Endothelial Cells (CMVEC)

Porcine CMVEC were isolated from newborn pig cerebral microvessels (60–300 µm) by collagenase-dispase digestion followed by the Percoll density gradient separation as described [33,36]. CMVEC were plated on Matrigel-coated plates and cultured in Dulbecco’s Modified Eagle Medium (DMEM) containing 20% fetal bovine serum (FBS), endothelial cell growth supplement (30 µg/mL), heparin (1 U/mL), and antibiotic/antimycotic mixture for 5–6 d. Primary adult human cerebral microvascular endothelial cells (CMVECs; ACBRI 376) from healthy brain cortex (passage 3) were purchased from Cell Systems (Kirkland, WA, USA) and cultured through passages 4–6 in Complete Classic Medium according to the manufacturer’s instructions. All experiments were conducted on confluent, quiescent cells after an overnight incubation in serum-depleted media.

### 2.7. In Vitro Model of BBB Permeability

Cerebral microvascular endothelial cells are essential components of the BBB, contributing to structural integrity and regulatory functions. We used primary CMVECs to investigate the effects of seizure-related oxidative stress, induced by seizure-related pro-inflammatory cytokine TNF-α, on endothelial barrier permeability during normothermic and hypothermic conditions. Monolayers of confluent, quiescent CMVEC from newborn pigs grown in transwells on Corning BioCoal Collagen inserts on 0.4 µm PET membranes (6.4 mm) were exposed to TNF-α (30 ng/mL) applied to the upper chamber (luminal side) during normothermia (37 °C) or hypothermia (30 °C and 24 °C) conditions for 1–6 h. Transendothelial electrical resistance TEER (Ohms) was measured using a Millicel electrical resistance system (Millicell-ERS, Millipore; Billerica, MA, USA) and normalized for the surface area of the cell monolayer as previously described [36]. To measure the paracellular permeability, 3 kDa dextran-conjugated Alexa Fluor 488 (1 µg/mL) was applied to the luminal side of CMVEC. Following the 6 h exposure to TNF-α, aliquots of media from the lower chamber (abluminal side) were collected, and Alexa Fluor 488 fluorescence (excitation/emission, 495/519 nm) was measured using a Synergy HT microplate reader (BioTek Instruments, Winooski, VT, USA).

### 2.8. ROS Detection

Dihydroethidium (DHE), which is oxidized to ethidium and oxyethidium and intercalates with DNA emitting red fluorescence, is commonly used to detect ROS production in tissues and cells [33,37]. Confluent quiescent primary porcine or human cerebral microvascular endothelial cells cultured on 12-well Costar plates were exposed to seizure-related pro-oxidants, pro-inflammatory cytokine TNF-α (30 ng/mL) or excitotoxic glutamate (1 mM), for 60 min at normothermia (37 °C) or hypothermia (30 °C or 24 °C). DHE (20 µM) was added to the media for the last 20 min of the incubation. The fluorescence of DNA-intercalated products of DHE oxidation (excitation/emission, 485/590 nm, [33]) was measured by a Synergy HT microplate reader (BioTek Instruments; Winooski, VT, USA) and normalized to the protein amount.

### 2.9. NADPH Oxidase Activity

NOX activity was measured as NADPH-dependent superoxide production using the lucigenin-enhanced luminescence assay [37]. Confluent quiescent CMVEC from newborn pigs were disrupted by sonication in ice-cold phosphate buffer containing protease inhibitors. Cell homogenates (~100 µg protein/sample) were incubated for 60 min at normothermia (37 °C) or hypothermia (24 °C) conditions with NADPH (100 µM) either alone or in the presence of TNF-α (15 ng/mL) or glutamate (0.2 mM). Lucigenin (50 µM), a chemiluminescent detector of the superoxide anion, was added for the last 20 min of the incubation. NADPH-enhanced lucigenin luminescence was measured by a Synergy HT microplate reader (BioTek Instruments, Winooski, VT, USA). NOX activity was expressed as luminescence units/100 µg protein.

### 2.10. In Vivo and In Vitro Apoptosis Detection: TUNEL Staining and DNA Fragmentation

DNA fragmentation and activation of the executioner caspase-3 are the key events in apoptosis. Apoptosis in cortical cerebral microvessels isolated from control and epileptic pigs was detected using the TUNEL-based apoptotic system kit to label DNA nicks (Trevigen Inc., Gaithersburg, MD, USA). Confluent quiescent porcine and human CMVECs were exposed to inflammatory (TNF-α, 30 ng/mL) or excitotoxic (glutamate, 1 mM) pro-oxidants for 3 h at normothermia (37 °C) or hypothermia (30 °C or 24 °C) conditions. Apoptotic DNA fragments were detected by ELISA assay (Roche Applied Science; Indianapolis, IN, USA) and visualized with 2,2’-azino-di (3-ethylbenzthiazolin-sulfonate) (ABTS) as described [33,36]. The reaction products of DNA fragmentation were measured spectrophotometrically at 405 nm and normalized to protein amount in the samples.

### 2.11. Western Blotting for Activated Caspase-3

CMVEC lysates were separated by SDS–PAGE and transferred to PVDF membranes. Membranes were blocked and incubated with cleaved caspase-3 (Asp175) primary antibodies (1:1000; Cell Signaling, Danvers, MA, USA). After washing, membranes were incubated with peroxidase-conjugated donkey anti-rabbit IgG (1:10,000; Sigma, St. Louis, MO, USA). Membranes were reprobed with monoclonal anti–β-actin (1:10,000; Sigma) as a loading control, followed by peroxidase-conjugated anti-mouse IgG (1:10,000, Sigma). Immunocomplexes were visualized using the Western Lightning chemiluminescence kit (PerkinElmer, Boston, MA, USA). The signals were recorded on X-ray film and quantified by digital densitometry. Because prestained MW markers on PVDF are not visible on film, membranes were aligned with the developed films, and MW marker bands were manually traced to verify protein size.

### 2.12. Materials

Bicuculline was purchased from Tocris (Minneapolis, MN, USA); pancuronium bromide from Astra Pharmaceutical Products (Westborough, MA, USA); dihydroethidium from Life Technologies (Thermo Fisher Scientific, Waltham, MA, USA); and cell culture reagents were from GE Healthcare Life Sciences (Pittsburg, PA, USA). All other reagents were purchased from Sigma (St. Louis, MO, USA).

### 2.13. Statistical Analysis

Data are presented as mean ± SD of absolute values or percentage of control. Data were analyzed by Student’s *t*-test and analysis of variance (ANOVA) for independent measurements. *p* values of < 0.05 were considered statistically significant.

## 3. Results

### 3.1. Selective Head Cooling Prevents BBB Damage in Epileptic Pigs

**Figure 1:**
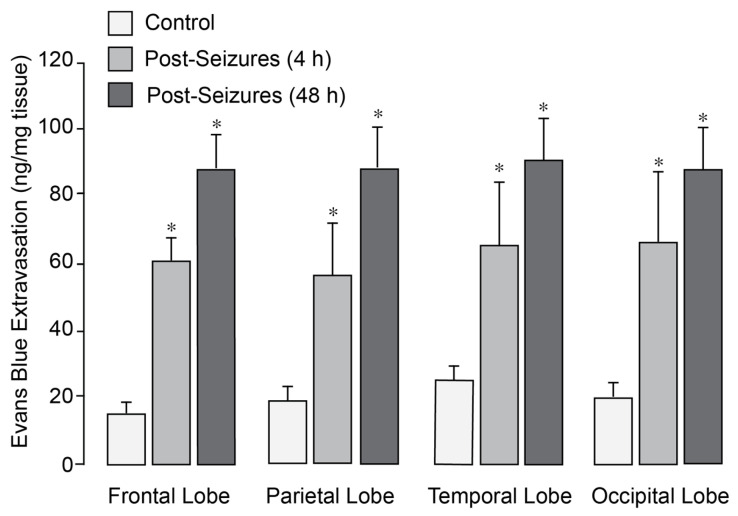
Epileptic seizures increase BBB permeability in newborn pigs. Seizures were induced by bicuculline (3 mg/kg, i.p.). BBB permeability was assessed by measuring Evans Blue extravasation in the frontal, parietal, temporal, and occipital lobes of the brain cortex in sham controls and postictal newborn pigs after 4 and 48 h of recovery following epileptic seizures. N = 6 pigs per group. Values are means ± SD. * *p* < 0.05, compared with the control group.

**Figure 2:**
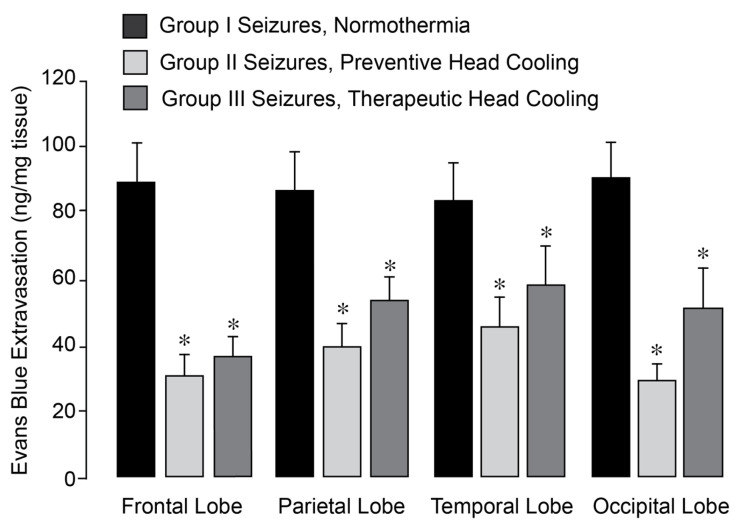
Selective head cooling improved the outcome of epileptic seizures on BBB permeability. Seizures were induced by bicuculline (3 mg/kg, i.p.) in control normothermic pigs (Group I) and in hypothermic pigs (Groups II and III). Selective head cooling was initiated either 30 min before bicuculline administration (preventive cooling, Group II) or 20 min afterwards (therapeutic cooling, Group III), resulting in mild hypothermia (core body temperature, 33.5 ± 0.1 °C; brain cortex temperature, 25.6 ± 0.3 °C). BBB permeability was assessed by measuring Evans Blue extravasation in the frontal, parietal, temporal, and occipital lobes of the brain cortex in postictal newborn pigs after 24 h of recovery following epileptic seizures. N = 6 pigs per group. Values are means ± SD. * *p* < 0.05, compared with the corresponding control values in normothermic pigs.

In normothermic epileptic pigs, seizures caused a 3- to 5-fold increase in BBB permeability, as evidenced by Evans Blue extravasation across the frontal, parietal, temporal, and occipital cortices 4–48 h after seizures (Figure 1). We previously demonstrated that application of head ice packs to neonatal pigs led to a reduction in brain cortex (25–26 °C) and core body (34–35 °C) temperatures [27]. Notably, in epileptic pigs treated with head cooling initiated before (Group II) or during seizures (Group III), the increase in BBB permeability across all regions was greatly attenuated (Figure 2).

**Figure 3:**
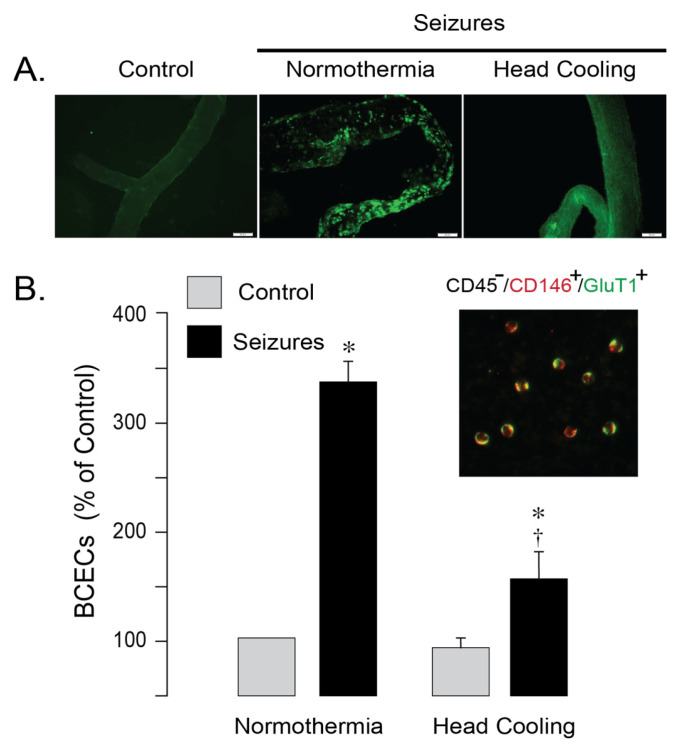
Selective head cooling reduced apoptosis in cerebral vessels induced by epileptic seizures. Seizures were induced by bicuculline (3 mg/kg, i.p.) in either normothermic or hypothermic pigs. Hypothermia (core body temperature, 33.5 ± 0.1 °C; brain cortex temperature, 25.6 ± 0.3 °C) was achieved by selective head cooling, initiated 30 min prior to bicuculline administration and maintained throughout the ictal period. (**A**): TUNEL staining of pial cerebral vessels isolated from control and epileptic pigs 6 h after bicuculline administration under normothermic and hypothermic conditions. (**B**): brain-derived circulating endothelial cells (BCECs), identified as CD45^−^/CD146^+^/GluT1^+^, are isolated from peripheral blood of control and epileptic pigs collected 6 h after bicuculline administration under normothermic and hypothermic conditions. N = 6 pigs per group. Values are means ± SD. * *p* < 0.05, compared with the corresponding control values in normothermic and hypothermic epileptic pigs. † *p* < 0.05, compared with the corresponding values in normothermic and epileptic pigs.

TUNEL staining revealed numerous apoptotic cells with fragmented DNA in cerebral vessels from normothermic epileptic pigs, whereas the number of TUNEL-positive cell was greatly attenuated in epileptic pigs treated by head cooling (Figure 3A). Peripheral blood mononuclear BCECs, characterized by co-expression of the common endothelial antigen (CD31) and the BBB-specific marker Glut1 (CD45^−^/CD31^+^/Glut1^+^) serve as the quantitative indicators of cerebral endothelial apoptosis [9,11,12]. In normothermic pigs, BCECs numbers greatly increased at 6 h after seizure induction (Figure 3B), indicating damage of the cerebral vascular endothelium. Selective head cooling markedly attenuated the seizure-induced elevation in BCECs (Figure 3B), consistent with improved endothelial survival and preservation of BBB integrity in epileptic pigs. Overall, these findings indicate that selective head cooling confers protection against seizure-induced cerebrovascular injury and disruption of the BBB.

### 3.2. Moderate Hypothermia Effectively Prevents Inflammation-Induced Disruption of the Endothelial Barrier

**Figure 4:**
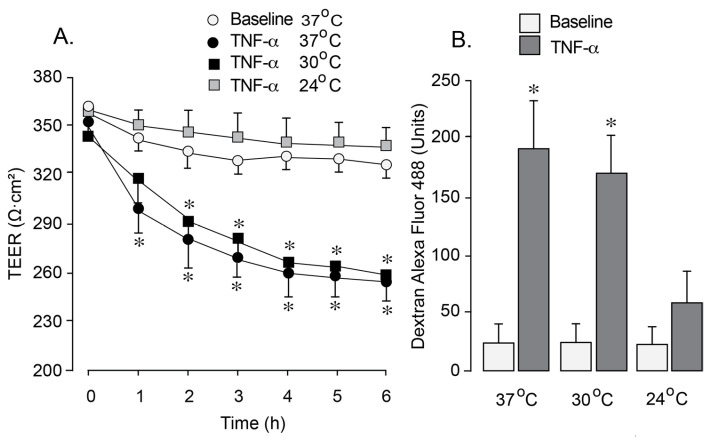
Effects of different levels of hypothermia on BBB permeability in an endothelial BBB model subjected to inflammation-induced oxidative stress. Confluent, quiescent CMVEC grown on semipermeable membranes in Costar Transwells were exposed to human recombinant TNF-α (30 ng/mL) for 1–6 h under normothermic conditions (37 °C) and two levels of hypothermia (30 °C and 24 °C). (**A**). TEER values (ohms, Ω) were measured using a Millicel electrical resistance system and normalized for the surface area of the cell monolayer. (**B**). Paracellular permeability of the endothelial monolayer to 3-kDa dextran-conjugated Alexa Fluor 488 was assessed after 6 h of incubation with TNF-α (30 ng/mL). Data represent the average of 4 experiments. Values are means ± SE. * *p* < 0.05 compared with the baseline values.

We utilized an in vitro blood–brain barrier (BBB) model to investigate the effects of hypothermia on endothelial barrier permeability under oxidative stress induced by inflammation. a common condition associated with epilepsy. Newborn pig cerebral microvascular endothelial cells (CMVEC) on semi-permeable membranes within Transwells inserts were exposed to pro-inflammatory cytokine TNF-α (30 ng/mL). Exposure to TNF-α at 37 °C for 1–6 h resulted in a reduction in transendothelial electrical resistance (TEER, Figure 4A) and an increased paracellular permeability to 3 kDa dextran (Figure 4B), suggesting BBB leakage. Exposure to TNF-α during mild hypothermia conditions (30 °C) resulted in a similar decline in TEER and a concomitant increase in paracellular permeability (Figure 4A,B). In contrast, lowering the incubation temperature to 24 °C completely prevented the TEER reduction and the increase in BBB paracellular permeability during the exposure to TNF-α (Figure 4A,B), suggesting a dramatic improvement of BBB integrity. These findings indicate that moderate hypothermia (<30 °C) has strong protective effects on endothelial BBB properties during inflammation.

### 3.3. Moderate Hypothermia Has Antioxidant and Antiapoptotic Effects in Brain Endothelial Cells Under Inflammatory and Excitotoxic Conditions

**Figure 5:**
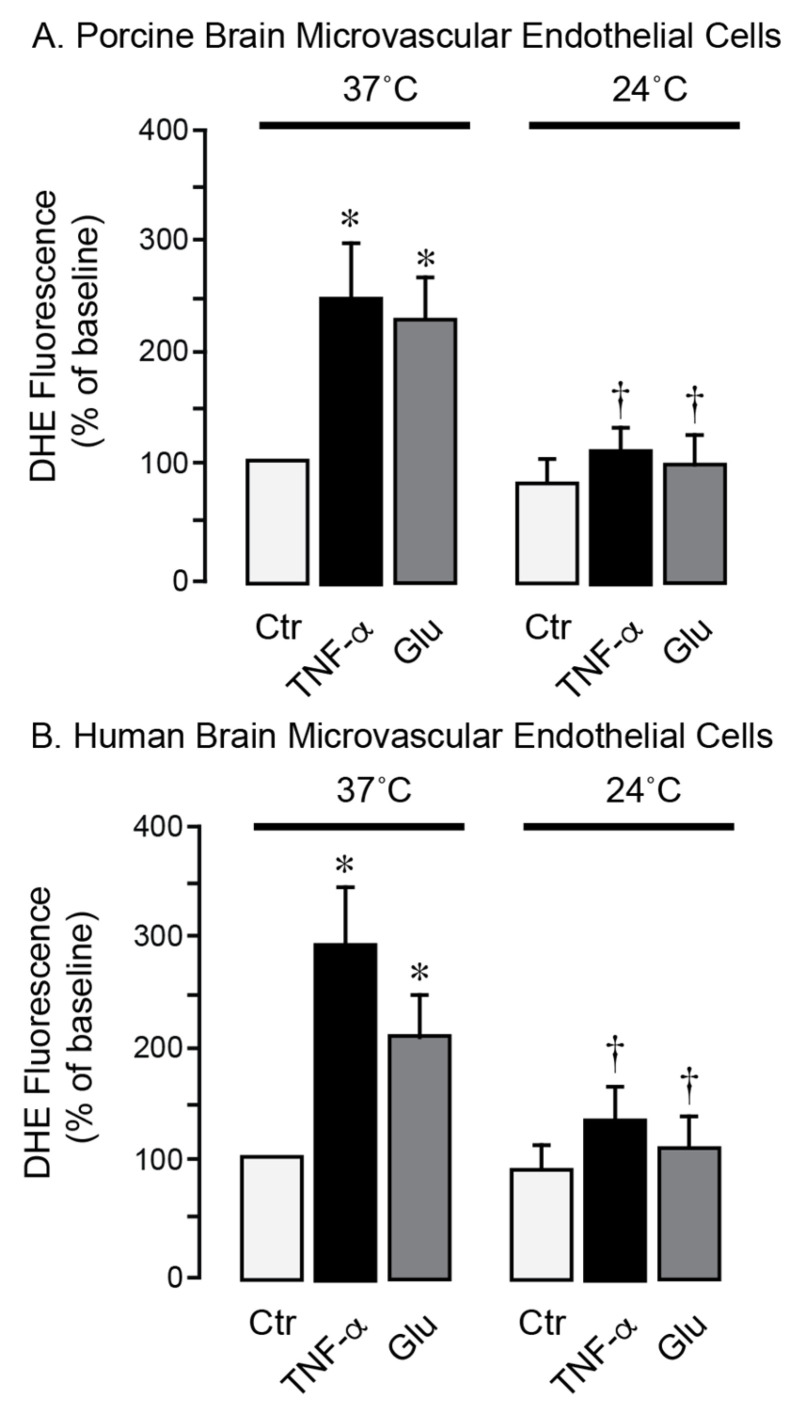
Hypothermia mitigates oxidative stress in porcine and human brain microvascular endothelial cells exposed to inflammatory and excitotoxic mediators. Primary microvascular endothelial cells derived from neonatal porcine brain (**A**) and adult human brain (**B**) were treated with TNF-α (30 ng/mL) or glutamate (1 mM) for 1 h during normothermic (37 °C) or hypothermic (24 °C) conditions. Reactive oxygen species were detected by DHE fluorescence and expressed as the % of the baseline control. Data represent the average of 4 independent experiments. Values are means ± SD. * *p* < 0.05 compared with the corresponding baseline value. ^†^ *p* < 0.05 compared with corresponding normothermia values.

**Figure 6:**
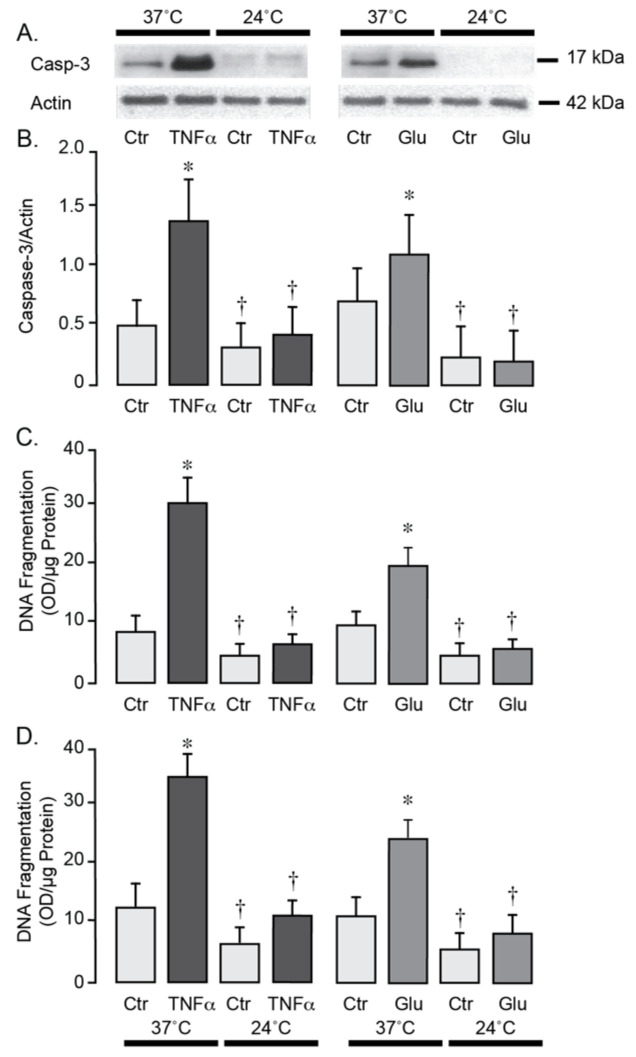
Hypothermia confers resistance to apoptosis in porcine (**A**–**C**) and human (**D**) cerebral microvascular endothelial cells during inflammation and excitotoxicity. Confluent quiescent piglet and human brain microvascular endothelial cells were treated with TNF-α (30 ng/mL) or glutamate (1 mM) for 3 h under normothermic (37 °C) or hypothermic (24 °C) conditions. Caspase-3 activation was assessed by Western immunoblotting (representative blot shown in (**A**)) and normalized to actin as the internal control (**B**). Apoptotic DNA fragmentation was quantified, normalized to protein amount, and expressed as (OD/µg protein) (**C**,**D**). Data represent the average of 4 independent experiments. Values are means ± SE. * *p* < 0.05 compared with the control values. † *p* < 0.05 compared with the corresponding normothermia values.

We used primary neonatal porcine and adult human brain endothelial cells to investigate the effects of hypothermia on cell survival during stress conditions relevant to seizures. Exposure of porcine and human brain endothelial cells to TNF-α (30 ng/mL) or excitotoxic glutamate (1 mM) for 1 h at 37 °C leads to a 2- to 4-fold increase in ROS production (Figure 5A,B). In contrast, lowering the incubation temperature to 24 °C completely suppressed the acute ROS responses to TNF-α and glutamate, consistent with antioxidant effects of moderate hypothermia. During normothermic conditions (37 °C), a 3-h exposure of porcine and human brain endothelial cells to TNF-α (30 ng/mL) or glutamate (1 mM) leads to apoptosis, as evidenced by caspase-3 activation (Figure 6A,B) and DNA fragmentation (Figure 6C,D). Remarkably, lowering the incubation media temperature to 24 °C completely prevented both key apoptotic responses induced by TNF-α or gluta-mate (Figure 6A–D). These findings suggest that moderate hypothermia (24 °C) efficiently mitigates oxidative stress and enhances the survival of porcine and human brain endothelial cells under inflammatory and excitotoxic conditions relevant to pathophysiology of epileptic seizures.

### 3.4. The Effects of Pharmacological Inhibitors and Hypothermia on NOX Activity in Brain Endothelial Cells

**Figure 7:**
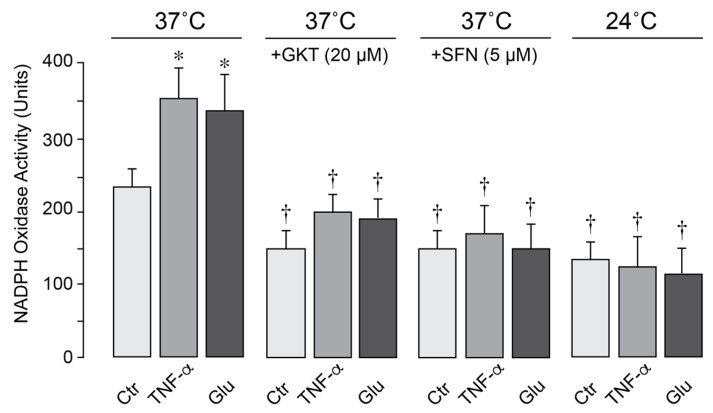
Hypothermia inhibits endothelial NOX activity. NOX activity was assessed by measuring superoxide production from NADPH (100 µM) using lucigenin-enhanced luminescence. Primary porcine CMVEC were either untreated (Ctr) or treated with TNF-α (15 ng/mL) or glutamate (0.2 mM) for 1 h under normothermic (37 °C) or hypothermic (24 °C) conditions in the absence or presence of NOX inhibitors setanaxib (GKT, 20 µM) and sulforaphane (SFN, 5 µM). Data represent the average of 4 independent experiments. Values are means ± SE. * *p* < 0.05 compared with the corresponding control values. † *p* < 0.05 compared with the corresponding values in the control normothermia group.

In porcine cerebral vascular endothelial cells, constitutive NOX activity was increased by TNF-α and glutamate following 1-h exposure at 37 °C (Figure 7). The selective NOX1/NOX4 inhibitor setanaxib (GKT137831, 20 µM) and antioxidant sulforaphane (5 µM) greatly reduced NOX activity under basal, inflammatory, excitotoxic conditions, confirming major contribution of NOX to oxidative stress. Notably, moderate hypothermia (24 °C) blocked baseline NOX activity and completely prevented its activation by TNF-α and glutamate (Figure 7). These findings indicate that moderate hypothermia mitigates oxidative stress caused by excitotoxic and inflammatory conditions by inhibiting endothelial NOX activity.

### 3.5. NOX Inhibitors Prevent BBB Disruption in the Epileptic Brain

**Figure 8:**
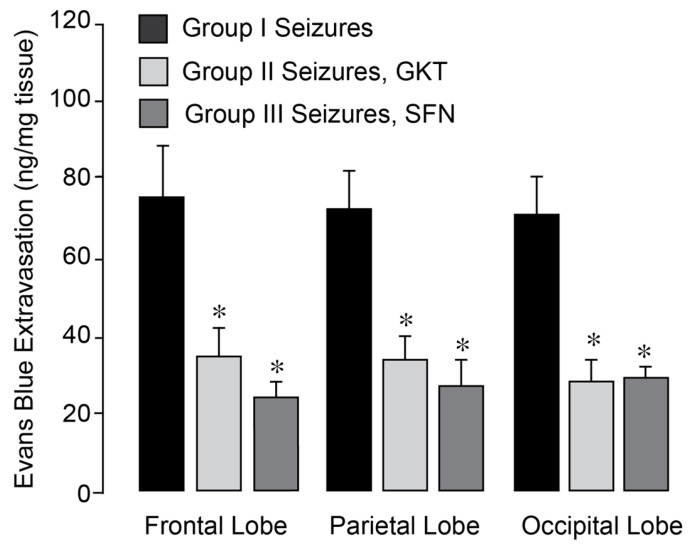
NOX inhibitors improved the outcome of epileptic seizures on BBB permeability. Seizures were induced by bicuculline (3 mg/kg, i.p.) in normothermic pigs, untreated (Group I) and pretreated with Nox inhibitors setanaxib (GKT, 5 mg/kg i.p, Group II) or sulforaphane (SFN, 0.4 mg/kg i.p, Group III). BBB permeability was detected by the Evans Blue extravasation in the frontal, parietal, and occipital lobes of the brain cortex 24 h after bicuculline administration. N = 4 pigs per group. Values are means ± SD. * *p* < 0.05, compared with the corresponding control values in untreated pigs.

We investigated whether pharmacological NOX inhibitors could attenuate the increase in BBB permeability in the epileptic brain. Epileptic seizures were induced in normothermic pigs, either untreated (Group I) or pretreated with GKT137831 (5 mg/kg i.p. Group II) or SFN (0.4 mg/kg i.p., Group III), administered 30 min prior to bicuculline administration. BBB permeability was assessed 24 h post-seizure by quantifying Evans Blue dye extravasation in the frontal, parietal, and occipital cortices of the brain. Both inhibitors effectively prevented BBB disruption in all examined cortical regions of the post-epileptic brain under normothermic conditions (Figure 8). These findings indicate that GKT137831 and sulforaphane are brain-permeable antioxidants and support the conclusion that NOX activation is the major source of oxidative stress driving sustained BBB leakage following seizures.

## 4. Discussion

Our study demonstrates that oxidative stress, primarily driven by NOX activity, plays a pivotal role in blood–brain barrier (BBB) disruption following neonatal epileptic seizures. Using a combination of in vivo and in vitro models, we show that both selective head cooling and pharmacological inhibition of NOX effectively preserve BBB integrity during seizure-induced oxidative stress.

### 4.1. Central Role of Oxidative Stress in Seizure-Induced Cerebrovascular Injury

Our previous work in a newborn pig model of glutamatergic epileptic seizures identified oxidative stress as a key driver of cerebral vascular dysfunction and impaired regulation of cerebral blood flow, extending into the delayed postictal period [27]. Notably, oxidative injury was concentrated in the cerebral cortical microvasculature, with limited involvement of the brain parenchyma. Endothelial cells emerged as especially vulnerable targets, primarily due to glutamate-induced excitotoxicity and pro-inflammatory cytokines. Importantly, we demonstrated that both classical antioxidants—such as Tiron and superoxide dismutase (SOD)—and endogenous heme oxygenase by-products, including carbon monoxide and bilirubin, were effective in reducing oxidative stress and preserving post-ictal neurovascular function [10]. Moreover, our findings suggest that selective head cooling during seizures provides substantial cerebrovascular protection [27]. These observations form the basis for a broader understanding of seizure-induced vascular pathology and open new avenues for therapeutic intervention.

### 4.2. Selective Head Cooling Preserved Blood–Brain Barrier Integrity in the Neonatal Epileptic Brain

Although the BBB integrity is essential for maintaining brain homeostasis during neonatal development, the impact of epileptic seizures on BBB permeability in neonates has remained largely unexplored. The present study provides novel evidence that epileptic seizures result in both acute and prolonged increases in BBB permeability across all major regions of the neonatal cerebral cortex. This sustained disruption of the BBB suggests a broader and more persistent vascular injury, with potential implications for neuronal health, immune activation, and long-term neurological outcomes. We report a novel and significant finding that selective head cooling during the ictal period preserves BBB integrity. In our neonatal pig model, applying ice packs directly to the skull during the ictal episode allows for selectively lowering cortex temperature to ~24–25 °C (moderate hypothermic range), while core body temperature remained within the mild hypothermic range (34–35 °C), which is considered safe for neonates [38,39]. However, total body cooling, the standard therapeutic approach for infants with moderate-to-severe asphyxia—which is often accompanied by seizures—reduces body temperature to the mild hypothermia range for up to 72 h but does not consistently achieve complete neurological recovery [38,39,40,41,42,43]. Selective head cooling was chosen in our experiments because the newborn brain generates approximately 70% of the body’s total heat, and prolonged systemic cooling may have physiologically harmful effects on vulnerable neonates [42,43]. Importantly, our results demonstrate that short-term head cooling restricted to the ictal period, achieving moderate hypothermia in the cooled region, provides sustained protection of BBB integrity for 4–48-h after seizures. These findings support both the safety and the translational potential of selective head cooling as a targeted neuroprotective strategy.

### 4.3. Circulating Brain-Derived Endothelial Cells and Cerebral Vascular Injury: Implications for Biomarkers Development

Cerebral vascular endothelial cells, which form the structural and functional basis of the BBB, are highly susceptible to oxidative stress-induced apoptotic injury [8,9,33]. The endothelial damage is characterized by the loss of intercellular junctions and cell detachment from the extracellular matrix, leading to appearance of brain-derived endothelial cells (BCECs) in peripheral blood [9]. In normothermic newborn pigs, elevated numbers of BCECs were detected in peripheral blood during both the acute and delayed postictal periods. This increase in BCECs coincided with the observed BBB leakage and likely reflects ongoing endothelial injury. The elevation of BCECs, apoptosis in cortical cerebral vessels, and BBB leakage were all markedly attenuated in pigs undergoing selective head cooling during seizures. These findings suggest that head cooling not only preserves cerebrovascular integrity but also reduces endothelial cell injury at both structural and systemic levels. The ability to monitor BCECs in peripheral blood thus represents a valuable, non-invasive approach for early detection, risk assessment, and therapeutic monitoring of cerebral vascular damage and BBB disruption in neonates experiencing neurological insults. Supporting its translational potential, our clinical observations demonstrate that BCECs elevation is also detectable in human newborns following neonatal hypoxia, asphyxia, and seizures and correlated with developmental outcome [11,12].

### 4.4. NOX Is a Central Mediator of Seizure-Induced Oxidative Injury and BBB Disruption

Our previous work using selective gene silencing methodology [32,33] demonstrated that NOX4-derived ROS are the dominant mediators of cerebral endothelial injury under both inflammatory and excitotoxic conditions relevant to seizures. Notably, NOX4 is constitutively expressed in cerebral vascular endothelial cells under non-stimulated conditions and is rapidly activated in response to tumor necrosis factor-alpha (TNF-α) and glutamate, leading to endothelial apoptosis. In contrast, other NOX isoforms such as NOX1 and NOX2 do not significantly contribute to the oxidative endothelial injury observed in seizure-related contexts [32,33].

In the current study, we provide new evidence that hypothermia suppresses endothelial NOX activity, reduces ROS levels, promotes endothelial cell survival, and preserves the BBB integrity. These findings reinforce our previous observation that NOX is the predominant source of oxidative stress responsible for seizure-induced BBB disruption. We further demonstrate that pharmacological inhibition of NOX4 with the selective inhibitor setanaxib effectively blocks ROS generation and prevents apoptosis in both porcine and human cerebral vascular endothelial cells exposed to TNF-α and glutamate. Similarly, the antioxidant sulforaphane, which also efficiently suppresses endothelial NOX activity [37], prevents apoptosis and promotes endothelial survival under inflammatory and excitotoxic conditions relevant to seizures. Importantly, in vivo administration of setanaxib or sulforaphane in normothermic pigs revealed that these compounds efficiently improve BBB integrity in the epileptic brain.

Our observations in the BBB model demonstrate that moderate hypothermia (24 °C) preserves endothelial cell viability and barrier integrity, whereas mild hypothermia (30 °C) fails to confer substantial protection under seizure-related oxidative stress conditions. Based on these findings, selective head cooling, which rapidly lowers cortical brain temperature to 24–25 °C while maintaining core body temperature at 34–35 °C, provides significant protection of the BBB during seizures and appears more effective for neuroprotection than total body cooling. Notably, this head cooling approach is effective whether applied before or during epileptic seizures, with rewarming initiated immediately after seizure termination.

## 5. Clinical Implications

Our findings have significant clinical implications for the management and prevention of cerebrovascular injury and BBB disruption in patients experiencing epileptic seizures, particularly in vulnerable populations such as neonates and individuals with drug-resistant epilepsy. First, the identification of NOX as a central mediator of oxidative stress in the cerebral endothelium highlights a specific and targetable molecular pathway contributing to seizure-induced vascular damage. This advances the current understanding of the pathophysiology of seizures beyond neuronal excitability, emphasizing the role of vascular and inflammatory components in disease progression and neurological outcomes.

Importantly, our demonstration that selective inhibition of NOX with setanaxib, as well as the use of sulforaphane, effectively reduces ROS production, prevents endothelial apoptosis, and preserves BBB integrity both in vivo and in vitro, provides a strong rationale for therapeutic targeting of endothelial oxidative stress. These agents, particularly setanaxib, which is already undergoing clinical testing in other oxidative stress-related disorders [44,45], may be repurposed for neuroprotective strategies in epilepsy care. Their brain-permeability and vascular-targeted effects are especially relevant for acute intervention following prolonged seizures or status epilepticus, where BBB breakdown can contribute to secondary neuronal injury and inflammation.

Furthermore, the ability of therapeutic hypothermia to inhibit NOX activity and preserve endothelial viability underscores its value as an adjunctive treatment, particularly in neonatal seizures or hypoxic–ischemic encephalopathy, where it is already in clinical use. Our observations in the endothelial BBB model demonstrate that moderate hypothermia (24–25 °C) preserves endothelial cell viability and barrier integrity, whereas mild hypothermia (30 °C) fails to confer substantial protection under seizure-related oxidative stress conditions. Based on these findings, selective head cooling, which rapidly lowers cortical brain temperature to 24–25 °C while maintaining core body temperature at 34–35 °C, provides significant protection of the BBB during seizures and appears more effective for neuroprotection than total body cooling. Notably, this approach is effective whether applied before or during epileptic seizures, with rewarming initiated immediately after seizure termination.

Taken together, these findings open new avenues for translational research and clinical trials aimed at reducing BBB damage and improving neurological outcomes following neonatal epileptic seizures. The convergence of hypothermia and pharmacological NOX inhibition on a common oxidative pathway suggests the possibility of synergistic or additive therapeutic effects. Targeting endothelial NOX may not only protect the BBB but also enhance the efficacy of antiepileptic treatments by maintaining drug permeability and reducing neuroinflammation. Importantly, monitoring BCECs in blood offers a valuable, non-invasive tool for early detection and longitudinal tracking of cerebral vascular injury and BBB disruption in neonates with neurological insults. Ultimately, this approach has the potential to shift current treatment paradigms by incorporating vascular protection as a core component of seizure management.

## 6. Conclusions

Collectively, our findings support the conclusion that endothelial NOX-mediated ROS production is a central mechanism of seizure-induced cerebrovascular damage and BBB disruption. Therapeutically targeting NOX through selective head cooling and pharmacological inhibitors, setanaxib and sulforaphane, represents a promising strategy for protecting the cerebral vasculature and mitigating BBB disruption associated with epileptic seizures.

**Study limitations include:** (1) a bicuculline-induced seizure model in newborn pigs may not fully reflect the heterogeneity of human neonatal seizures; (2) evaluation of BBB permeability confined to a 4–48 h postictal window, limiting assessment of longer-term outcomes; (3) use of an endothelial monoculture BBB model that does not incorporate other neurovascular unit components such as astrocytes and pericytes; and (4) uncertain translational applicability of the selective head cooling protocol to human neonates. These limitations, however, do not detract from the central finding that both NOX inhibition and selective head cooling preserve BBB integrity following neonatal seizures.

## Data Availability

The original contributions presented in this study are included in the article. Further inquiries can be directed to the corresponding author.
